# Aqueous Extract of Gumiganghwal-tang, a Traditional Herbal Medicine, Reduces Pulmonary Fibrosis by Transforming Growth Factor-β1/Smad Signaling Pathway in Murine Model of Chronic Asthma

**DOI:** 10.1371/journal.pone.0164833

**Published:** 2016-10-14

**Authors:** Woo-Young Jeon, In-Sik Shin, Hyeun-Kyoo Shin, Seong Eun Jin, Mee-Young Lee

**Affiliations:** 1 K-herb Research Center, Korea Institute of Oriental Medicine, Daejeon, Republic of Korea; 2 College of Veterinary Medicine, Chonnam National University, Gwangju, Republic of Korea; Forschungszentrum Borstel Leibniz-Zentrum fur Medizin und Biowissenschaften, GERMANY

## Abstract

Gumiganghwal-tang is a traditional herbal prescription that is used widely for the treatment of the common cold and inflammatory diseases in Korea and other Asian countries. In this study, we investigated the protective effects of a Gumiganghwal-tang aqueous extract (GGTA) against airway inflammation and pulmonary fibrosis using a mouse model of chronic asthma. Chronic asthma was modeled in BALB/c mice via sensitization/challenge with an intraperitoneal injection of 1% ovalbumin (OVA) and inhalation of nebulized 1% OVA for 4 weeks. GGTA (100 mg/kg or 200 mg/kg) was also administered by oral gavage once a day for 4 weeks. We investigated the number of inflammatory cells, production of T-helper type 2 (Th2) cytokines, chemokine and the total transforming growth factor-β1 (TGF-β1) in bronchoalveolar lavage fluid (BALF); the levels of immunoglobulin E (IgE) in the plasma; the infiltration of inflammatory cells in lung tissue; and the expression of TGF-β1, Smad-3, and collagen in lung tissue. Our results revealed that GGTA lowered the recruitment of inflammatory cells (particularly, lymphocyte); and decreased the production of Th2 cytokines, chemokine and total TGF-β1; and attenuated the levels of total and OVA-specific IgE; and decreased the infiltration of inflammatory cells. Moreover, GGTA significantly reduced the expression of TGF-β1 and Smad-3, and lowered collagen deposition. These results indicate that GGTA reduces airway inflammation and pulmonary fibrosis by regulating Th2 cytokines production and the TGF-β1/Smad-3 pathway, thus providing a potential treatment for chronic asthma.

## Introduction

Allergic asthma is a chronic inflammatory disease of the lung associated with excessive airway infiltration of inflammatory cells into lung tissues, elevated levels of allergen-specific immunoglobulin E (IgE), overexpression of T-helper type 2 (Th2) cytokines and chemokine including IL-4, IL-5, IL-13, and eotaxin [1]. Gradually, chronic asthma spanning over a long period is characterized by pulmonary inflammation, subepithelial/peribranchial fibrosis, and collagen deposition [2].

Th2-type cytokines, which are secreted by Th2 cells, play a central role in the pathogenesis of allergic asthma by regulating IgE production, the release of a variety of inflammatory mediators, and the differentiation and activation of eosinophils [3]. Th2 cytokines are also associated with a growth factor, the transforming growth factor-β1 (TGF-β1), which is a profibrotic cytokine that is thought to play an important role in chronic asthma [4]. TGF-β1 is a master regulator of immune responses resulting in fibrosis via the deposition of collagen [5]. It initiates canonical and noncanonical pathways to exert multiple biological effects. Among them, Smads are key proteins that are recognized as a major pathway of TGF-β1 signaling in progressive renal fibrosis [6]. Smads are categorized into three subfamilies according to function: pathway-restricted Smads (Smad2 and Smad3), common mediator Smad (Smad4), and inhibitory Smads (Smad6 and Smad7) [7]. In particular, Smad-3 is highly activated during fibrogenesis and is then translocated to nuclei, where it induces expression of TGF-β1 target genes, such as the gene that encodes collagen [6, 8]. According to a previous study, the TGF-β1/Smad signaling pathway is one of the important mechanisms involved in the development of asthma [9].

Herbal medicines have long been used widely in many countries around the world, and are composed of various herbs with ubiquitous pharmacological activities [10]. The traditional herbal medicine Gumiganghwal-tang (known as Jiu Wei Qianghuo tang in China and Kumi-Kyokatsu-to in Japan) is composed of different herbs and has been used for the treatment of the common cold, headache, arthralgia, and fever in Asian countries [11]. Many studies have demonstrated that Gumiganghwal-tang exerts anti-inflammatory [12, 13] and neuroprotective effects [11] *in vitro* and in *vivo*. However, the mechanism of regulation of the antiasthmatic and antifibrotic activity of this medicine in OVA-induced asthma remains unknown. Therefore, experimental studies were conducted to investigate whether a Gumiganghwal-tang aqueous extract (GGTA) has antiasthma and antifibrotic effects on airway inflammation and pulmonary fibrosis in an OVA-induced chronic asthma model.

## Materials and Methods

### Reagents

The chopped crude herb of Gumiganghwal-tang was purchased from Omniherb (Yeongcheon and Kunwi, Korea) and HMAX (Jeongseon, Korea and China), and GGTA was prepared from a mixture of herbs in our laboratory (Table 1). GGTA was obtained via extraction in distilled water at 100°C for 120 min. The extract was evaporated to dryness and freeze dried (yield, 22.80%). The extracted Gumiganghwal-tang powder was stored at 4°C. Montelukast (Mon) was purchased from Sigma-Aldrich (SML0101; St. Louis, MO, USA) and was used as the positive control drug. Other reagents were as follows: ovalbumin (OVA, albumin from chicken egg white; A5503; Sigma-Aldrich), aluminum hydroxide (239186; Sigma-Aldrich), pentobarbital (Hanlim Pharm. Co., Seoul, Korea), enzyme-linked immunosorbent assay (ELISA) kits (R&D Systems, Minneapolis, MN, USA; BioSource International, Camarillo, CA; and BioLegend Corp., San Diego, CA, USA), hematoxylin solution (MHS16; Sigma-Aldrich), eosin Y solution, (HT110132, Sigma-Aldrich), and anti-TGF-β1 (ab64715; Abcam, Cambridge, UK & Cambridge, MA, USA), anti-Smad-3 (sc-101154; Santa Cruz Biotechnology, Santa Cruz, CA, USA), and anti-β-actin (#4967S; Cell Signaling Technology, Danvers, MA, USA) antibodies.

**Table 1:** Composition of Gumiganghwal-tang.

Latin name	Amount (g)	Supplier	Source
Osterici Radix	5.625 (15%)	HMAX	China
Saposhnikoviae Radix	5.625 (15%)	HMAX	China
Cnidii Rhizoma	4.5 (12%)	Omniherb	Yeongcheon, Korea
Angelicae Dahuricae Radix	4.5 (12%)	Omniherb	Yeongcheon, Korea
Atractylodis Rhizoma	4.5 (12%)	HMAX	China
Scutellariae Radix	4.5 (12%)	HMAX	Jeongseon, Korea
Rehmanniae Radix Crudus	4.5 (12%)	Omniherb	Kunwi, Korea
Asiasari Radix	1.875 (5%)	HMAX	China
Glycyrrhizae Radix et Rhizoma	1.875 (5%)	HMAX	China
Total amount	37.5 (100%)		

### Animals

BALB/c female mice (6 weeks of age) were purchased from Orientbio Inc. (Seoul, Korea) and acclimated in an animal-care facility for 1 week. The mice were housed in environmentally controlled and specific pathogen-free conditions (22°C, 12 h light/12 h dark cycle) and were provided with water and standard chow *ad libitum*. Mice were randomly divided into five groups (eight animals/group): NC (normal control group: phosphate buffered saline (PBS)-sensitization/challenge + oral gavage of PBS), OVA (OVA-sensitization/challenge group + oral gavage of PBS), Mon (OVA-sensitization/challenge group + oral gavage of montelukast at 30 mg/kg), GGTA-100 (OVA-sensitization/challenge group + oral gavage of GGTA at 100 mg/kg), and GGTA-200 (OVA-sensitization/challenge group + oral gavage of GGTA at 200 mg/kg). All experimental procedures were carried out in accordance with the National Institutes of Health Guidelines for the Care and Use of Laboratory Animals and were approved by the Institutional Animal Care and Use Committee of the Korea Institute of Oriental Medicine. Animal handling was carried out in accordance with the dictates of the National Animal Welfare Law of Korea.

### Animal model

**Fig 1:**
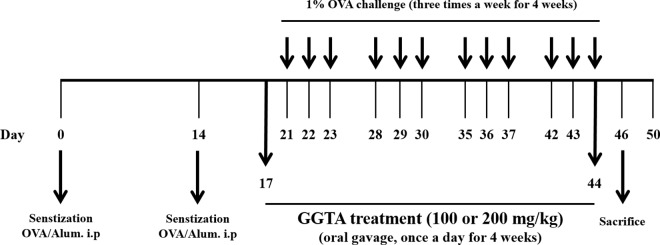
Flow diagram of the experimental procedure: mouse model of chronic asthma. OVA, ovalbumin; OVA/Alum, ovalbumin/aluminum hydroxide; i.p., intraperitoneal injection; GGTA, Gumiganghwal-tang aqueous extract.

The design of this experiment outlined in Fig 1. OVA-induced mice were sensitized on days 0 and 14 via an intraperitoneal injection of OVA (20 μg) emulsified with 2 mg of aluminum hydroxide in 200 μL of PBS (pH 7.4). One week after the second sensitization (on day 21), the mice underwent an airway challenge with OVA (1%, w/v, in PBS) for 1 h using an ultrasonic nebulizer (NE-U12; Omron Corp., Tokyo, Japan) three times a week for 4 weeks. Each day during the 4 weeks of the airway challenge, GGTA (100 and 200 mg/kg) was dissolved in PBS and was prepared fresh daily before each administration. GGTA was administered by gavage to mice at doses of 100 or 200 mg/kg once daily from days 17 to 44. The normal- and positive-control groups were administered PBS or Mon orally (30 mg/kg in PBS), respectively. Montelukast, a cysteinyl-leukotriene receptor antagonist, is used commercially as a maintenance treatment for asthma and to relieve allergic symptoms [14, 15].

Forty-eight hours after the last challenge, the mice were sacrificed by intraperitoneal injection of pentobarbital (50 mg/kg), and tracheostomy was performed. To obtain BALF, ice-cold PBS (0.5 mL) was infused into the lung and withdrawn via tracheal cannulation three times (total volume, 1.5 mL). The number of total inflammatory cells was assessed by counting cells in at least 5 squares of a hemocytometer after exclusion of dead cells by Trypan blue staining. To identify the differential cell counts, 100 μL of BALF was centrifuged onto slides using a cytospin machine (Hanil Science Industrial, Seoul, Korea) at 1,500 rpm for 10 min at 4°C. The slides were dried and the cells were fixed and stained using the Diff-Quik^®^ staining reagent (B4132-1A; IMEB Inc., Deerfield, IL), according to the manufacturer’s instructions. The cell supernatant obtained from BALF was stored at –70°C for biochemical analysis. Plasma samples were obtained from the mice via the inferior vena cava. Plasma was collected via centrifugation (3,000 rpm for 10 min at 4°C) and subsequently stored at −70°C. Lung tissue was homogenized (1/10 w/v) in tissue lysis/extraction reagent containing protease inhibitor cocktail using a homogenizer (IKA T10 Basic Ultra Turrax; IKA Works, Staufen, Germany). Homogenates were centrifuged at 12,000 rpm for 20 min at 4°C. Total protein concentrations in the supernatant fractions were measured using Bradford reagent (#500–0006; Bio-Rad Laboratories, Inc., Hercules, CA, USA) and subsequently stored at −70°C.

### ELISA

The levels of IL-4, IL-5, IL-13, eotaxin, total TGF-β1, and IgE in BALF and plasma were measured using ELISA kits according to the manufacturer’s protocols, as described previously [2]. The detection limit of mouse IL-4 is 2 pg/mL, mouse IL-5 is 7 pg/mL, mouse IL-13 is 1.5 pg/mL, mouse eotaxin is 3.6 pg/mL, mouse total TGF-β1 is 4.6 pg/mL, and mouse IgE is 100 pg/mL.

### Histological examination

After BALF samples were obtained, the removed-lung tissue was fixed in 4% paraformaldehyde (sc-281692; Santa Cruz Biotechnology). Tissues were embedded in paraffin, sectioned at 4 μm thickness, and stained with hematoxylin and eosin (H&E) solution to estimate inflammatory cell accumulation. Collagen accumulation was examined in the lung sections with the collagen-specific stain Sirius red-picric solution. The sections were washed rapidly with acetic acid, and stained sections were observed and photographed under a light microscope (H&E stain; at a magnification of × 200 and Sirius red stain; at a magnification of × 100, respectively). Quantitative analysis for airway inflammation and collagen accumulation was evaluated using a MetaMorph Offline version 7.7.0.0 image analysis software (Molecular Devices Inc., Sunnyvale, CA, USA).

### Collagen assay

The deposition of collagen in the lung tissue was measured using Sircol collagen assay kits (#S1000; Biocolor, Carrickfergus, UK) according to the manufacturer’s protocols. Total proteins were determined using a protein assay reagent. The results were expressed as μg/mg of protein.

### Western blotting

Equal amounts of total lung protein (30 μg) were heated at 100°C for 5 min, loaded onto 8% sodium dodecyl sulfate polyacrylamide gel electrophoresis gels, and electrophoresed. The proteins were then transferred to an Immobilon-P polyvinylidene difluoride (PVDF) membrane (IPVH00010; Millipore Corporation, Bedford, MA, USA) (at 100 V for 90 min). The membrane was blocked for 1 h with Tris-buffered saline containing 0.05% Tween-20 (TBST) plus 5% skim milk, followed by incubation with anti-TGF-β1 (1:1000 dilution), anti-Smad-3 (1:1000 dilution), and anti-β-actin (1:1000 dilution) antibodies overnight at 4°C. The membrane was washed three times with TBST at intervals of 10 min and then incubated with a horseradish peroxidase (HRP)-conjugated secondary antibody (TGF-β1, anti-mouse; Smad-3, anti-mouse; and β-actin, anti-rabbit; 1:3000 dilution, respectively) (Jackson ImmunoResearch, West Grove, PA, USA) for 1 h at room temperature. The membrane was washed three times with TBST at intervals of 10 min and developed using the SuperSignal^®^ West Femto Maximum Sensitivity Substrate (ultrasensitive enhanced chemiluminescent substrate) (NCI34095KR; Thermo Fisher Scientific Inc., Waltham, MA, USA). Subsequently, membranes were photographed and, for quantitative analyses, densitometric band values were determined using the commercially available ChemiDoc^TM^ XRS^+^ Imaging System (Bio-Rad Laboratories).

### Statistical analysis

All data are presented as the mean ± standard error of the mean (SEM). Statistical significance was determined using analysis of variance (ANOVA) followed by a multiple comparison test with Bonferroni adjustment. *P* values of < 0.05 were considered statistically significant.

## Results

### Effects of GGTA on the recruitment of inflammatory cells in BALF

**Fig 2:**
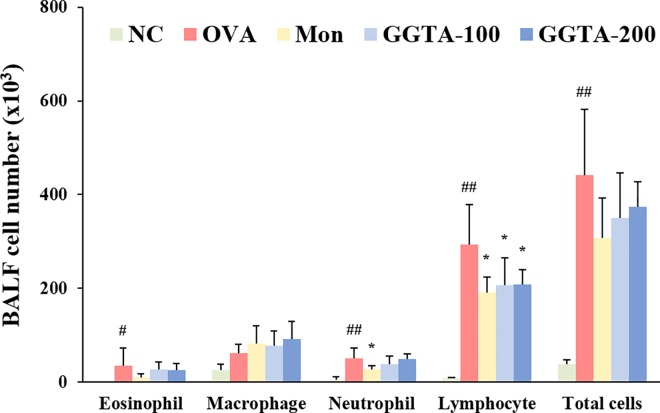
Effect of GGTA on the recruitment of inflammatory cells in the bronchoalveolar lavage fluid (BALF). BALF cell were isolated by cytospin and stained with Diff-Quik^®^ staining reagent. Numbers of cell were determined using a light microscope to count cells. NC, normal-control mice (vehicle group, PBS); OVA, OVA-induced mice (control group); Mon, OVA-induced mice + montelukast (30 mg/kg) (positive-control group); GGTA-100, OVA-induced mice + GGTA (100 mg/kg); GGTA-200, OVA-induced mice + GGTA (200 mg/kg). Values are expressed as means ± SEM (n = 8 per group). Significant differences at ^#^*P* < 0.05 and ^##^*P* < 0.01 compared with the NC group, respectively. Significant differences at ^*^P < 0.05 compared with the OVA-induced group.

To identify the inflammatory cells, we counted the number of total cells such as eosinophil, macrophage, neutrophil, and lymphocyte in BALF. As shown in Fig 2, the number of inflammatory cells in BALF were significantly elevated in the OVA-induced group compared with the NC group. However, The OVA-induced mice treated with GGTA (100 and 200 mg/kg) reduced the number of total cells, similar to Mon-treated group (particularly, lymphocyte).

### Effects of GGTA on the production of IL-4, IL-5, IL-13, and eotaxin in BALF

**Fig 3:**
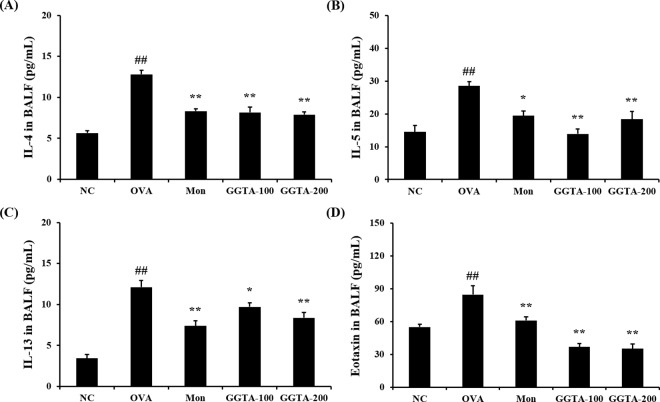
Effect of GGTA on the production of Th2 cytokines and chemokine in the BALF. Individual data were obtained using ELISA. Th2 cytokines: (A) IL-4, (B) IL-5, and (C) IL-13; chemokine (D) eotaxin. NC, normal-control mice (vehicle group, PBS); OVA, OVA-induced mice (control group); Mon, OVA-induced mice + montelukast (30 mg/kg) (positive-control group); GGTA-100, OVA-induced mice + GGTA (100 mg/kg); GGTA-200, OVA-induced mice + GGTA (200 mg/kg). Values are expressed as means ± SEM (n = 8 per group). Significant differences at ^##^*P* < 0.01 compared with the NC group. Significant differences at ^*^P < 0.05 and ^**^P < 0.01 compared with the OVA-induced group, respectively.

To determine whether GGTA influenced Th2 cytokines and chemokine production into BALF, we evaluated the levels of IL-4, IL-5, IL-13, and eotaxin in BALF. The levels of IL-4, IL-5, IL-13, and eotaxin were significantly elevated in the OVA-induced group (12.81 ± 1.33, 28.54 ± 3.86, 12.11 ± 2.27, and 84.70 ± 23.49 pg/mL, respectively) compared with the NC group (5.67 ± 0.63, 14.49 ± 5.27, 3.42 ± 1.24, and 55.03 ± 6.92 pg/mL, respectively). However, treatment with GGTA (100 mg/kg: 8.17 ± 1.74, 13.91 ± 4.48, 9.71 ± 1.45, and 36.87 ± 8.55 pg/mL; 200 mg/kg: 7.89 ± 0.97, 18.42 ± 6.77, 8.38 ± 1.81, and 35.20 ± 11.87 pg/mL, respectively), as well as treatment with Mon (8.32 ± 0.72, 28.54 ± 3.86, 7.39 ±1.74, and 61.10 ± 8.68, respectively) reduced the levels of Th2 cytokines, including IL-4 (Fig 3A), IL-5 (Fig 3B), and IL-13 (Fig 3C), and of chemokines, such as eotaxin (Fig 3D), compared with the OVA-induced group. In the IL-5, administration of GGTA at a dose of 100 mg/kg inhibited IL-5 production more than treatment with GGTA 200 mg/kg in the BALF. Commonly, herbal prescription is complex of several herbal extract, there was several activity among the single components. It seems that each component interaction affect negative effects. Therefore, sometimes lower dosage of herbal extract is more effective than higher dosage.

### Effects of GGTA on the levels of total and OVA-specific IgE in the plasma

**Fig 4:**
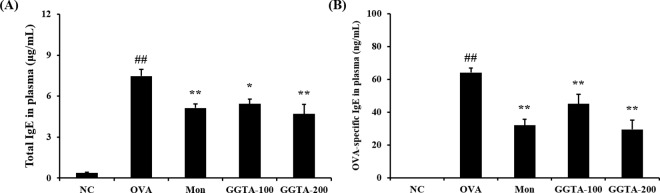
Effects of GGTA on the levels of total and OVA-specific IgE in the plasma. Individual data were obtained using ELISA. (A) Total IgE and (B) OVA-specific IgE. NC, normal-control mice (vehicle group, PBS); OVA, OVA-induced mice (control group); Mon, OVA-induced mice + montelukast (30 mg/kg) (positive-control group); GGTA-100, OVA-induced mice + GGTA (100 mg/kg); GGTA-200, OVA-induced mice + GGTA (200 mg/kg). Values are expressed as means ± SEM (n = 8 per group). Significant differences at ^##^*P* < 0.01 compared with the NC group. Significant differences at ^*^P < 0.05 and ^**^P < 0.01 compared with the OVA-induced group, respectively.

To investigate further the alterations in the adaptive immune response resulting from immunization with GGTA, we investigated the levels of total and OVA-specific IgE in the plasma. As shown in Fig 4, the level of total and OVA-specific IgE in the plasma was much higher in the OVA-induced group (7.48 ± 1.26 μg/mL and 64.22 ± 7.43 ng/mL, respectively) than it was in the NC group (0.38 ± 0.16 μg/mL and not detected, respectively). In contrast, the GGTA-treated groups (100 and 200 mg/kg) exhibited significantly decreased levels of total (100 mg/kg: 5.43 ± 1.00 μg/mL; and 200 mg/kg: 4.70 ± 1.92 μg/mL, respectively) and OVA-specific (100 mg/kg: 45.13 ± 15.21 ng/mL; and 200 mg/kg: 29.50 ± 15.08 ng/mL, respectively) IgE compared with the OVA-induced group, similar to what was observed in the Mon-treated group (5.14 ± 0.84 μg/mL and 31.99 ± 9.99 ng/mL, respectively).

### Effects of GGTA on the infiltration of inflammatory cells in lung tissues

**Fig 5:**
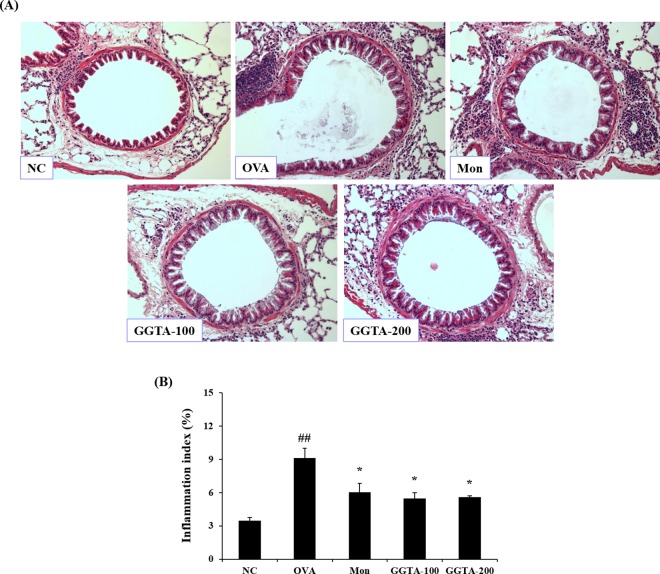
Effects of GGTA on the infiltration of inflammatory cells in lung tissues. Lung tissues were stained with (A) hematoxylin and eosin (H&E) solution for histological examination (magnification, 200×). (B) Inflammation index were determined using an image analyzer. Representative photomicrographs of lung sections are shown. NC, normal-control mice (vehicle group, PBS); OVA, OVA-induced mice (control group); Mon, OVA-induced mice + montelukast (30 mg/kg) (positive-control group); GGTA-100, OVA-induced mice + GGTA (100 mg/kg); GGTA-200, OVA-induced mice + GGTA (200 mg/kg). Values are expressed as means ± SEM (n = 3 per group). Significant differences at ^##^*P* < 0.01 compared with the NC group. Significant differences at ^*^P < 0.05 compared with the OVA-induced group, respectively.

The development of airway inflammation was evaluated by histopathological analysis of lung lesions. To perform a histopathological analysis of lung lesions, we stained lung sections with H&E. In the OVA-induced group, marked infiltration of inflammatory cells into lung tissues was observed, compared with the NC group. The OVA-induced mice treated with GGTA (100 and 200 mg/kg) exhibited markedly attenuated infiltration of inflammatory cells similar to the Mon-treated group, producing slightly better results (Fig 5A and 5B).

### Effects of GGTA on total TGF-β1 production and collagen deposition in BALF and lung tissues

**Fig 6:**
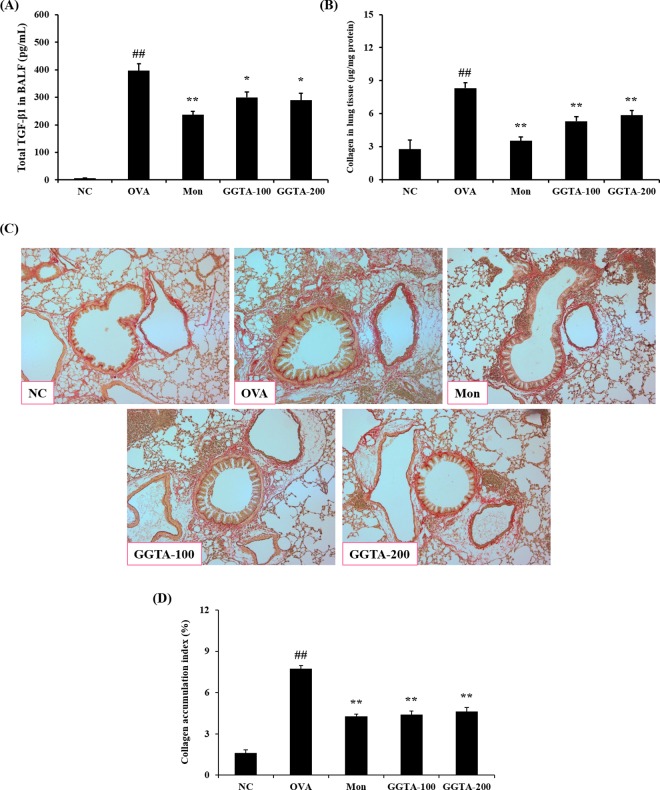
Effects of GGTA on total TGF-β1 production and collagen deposition in BALF and lung tissues. Individual data were obtained using ELISA, a collagen assay, Sirius red staining (magnification, 100×) and quantitative analysis, respectively. (A) Total TGF-β1, (B and C) collagen and (D) collagen accumulation index were determined using an image analyzer. NC, normal-control mice (vehicle group, PBS); OVA, OVA-induced mice (control group); Mon, OVA-induced mice + montelukast (30 mg/kg) (positive-control group); GGTA-100, OVA-induced mice + GGTA (100 mg/kg); GGTA-200, OVA-induced mice + GGTA (200 mg/kg). Values are expressed as means ± SEM (n = 8 per group, Sirius red staining; n = 3 per group). Significant differences at ^##^*P* < 0.01 compared with the NC group. Significant differences at ^*^P < 0.05 and ^**^P < 0.01 compared with the OVA-induced group, respectively.

To confirm the correlation between the activation of TGF-β1 and collagen synthesis, we evaluated total TGF-β1 production and collagen deposition in BALF and lung tissues. As shown in Fig 6A and 6B, total TGF-β1 production and collagen deposition were significantly higher in the OVA-induced mice (397.29 ± 65.86 pg/mL and 8.33 ± 1.39 μg/mg of protein, respectively) than they were in the NC mice (5.70 ± 3.77 pg/mL and 2.77 ± 2.20 μg/mg of protein, respectively). However, the GGTA-treated mice (100 and 200 mg/kg) had a significantly lower production of total TGF-β1 (100 mg/kg: 300.31 ± 47.40 pg/mL; and 200 mg: 289.26 ± 66.97 pg/mL, respectively) and deposition of collagen (100 mg/kg: 5.32 ± 1.17 μg/mg protein; and 200 mg/kg: 5.85 ± 1.21 μg/mg protein, respectively) compared with the OVA-induced mice, similar to what was observed in the Mon-treated mice (236.48 ± 33.72 pg/mL and 3.53 ± 0.97 μg/mg protein, respectively). For confirmation of the results of Fig 6B, the accumulation of collagen was evaluated by histopathological analysis of lung lesions and we stained lung sections with Sirius red. In the OVA-induced group, collagen fibers surrounding the central vein area, and overt signs of bronchiolar fibrosis was observed, compared with the NC group. The OVA-induced mice treated with GGTA (100 and 200 mg/kg) markedly inhibited accumulation of collagen similar to Mon-treated group (Fig 6C and 6D).

### Effects of GGTA on the expression of TGF-β1 and Smad-3 in lung tissues

**Fig 7:**
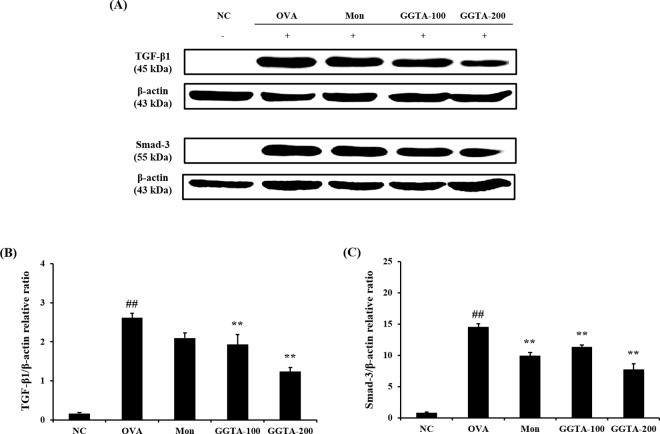
Effects of GGTA on the expression of TGF-β1 and Smad-3 in lung tissues. Lung tissues were homogenized and TGF-β1/Smad-3 protein expression was determined using western blot analysis. (A) Expression of TGF-β1 and Smad-3; relative ratio: (B) relative levels of TGF-β1 expression and (C) relative levels of Smad-3 expression. The β-actin protein was used as the loading control for quantitative analysis. The relative ratios of protein expression were normalized to β-actin and are represented in quantitative graphs, respectively. NC, normal-control mice (vehicle group, PBS); OVA, OVA-induced mice (control group); Mon, OVA-induced mice + montelukast (30 mg/kg) (positive-control group); GGTA-100, OVA-induced mice + GGTA (100 mg/kg); GGTA-200, OVA-induced mice + GGTA (200 mg/kg). Values are expressed as means ± SEM (n = 8 per group). Significant differences at ^##^*P* < 0.01 compared with the NC group. Significant differences at ^**^P < 0.01 compared with the OVA-induced group, respectively.

To investigate the expression of the active TGF-β1 signaling pathway in the lung, we examined the expression of TGF-β1 and of the intracellular effector Smad (Smad-3). As shown in Fig 7A, an increase in the expression of TGF-β1 and Smad-3 was observed during allergen challenge, an increase in the expression of TGF-β1 and Smad-3 was observed following allergen challenge relative to the NC mice. The administration of GGTA (100 and 200 mg/kg) and Mon both markedly decreased the expression of TGF-β1 and Smad-3. The relative ratios of TGF-β1/ and Smad-3/β-actin were significantly increased in the OVA-induced group compared with the NC group (*P* < 0.01). In contrast, the GGTA-treated groups (100 and 200 mg/kg) exhibited a significantly reduced relative ratio of TGF-β1/ and Smad-3/β-actin compared with the OVA-induced group (*P* < 0.01) (Fig 7B and 7C).

## Discussion

The aim of the present study was to investigate the effects of GGTA as an agent that can be used to improve the asthma-related immunological changes occurring during the airway inflammation and pulmonary fibrosis that follow chronic asthma in a mouse model. Our findings indicated that GGTA attenuated the accumulation of inflammatory cells into airway and lung tissue, with decreased production of Th2 cytokines, chemokine, total TGF-β1 and, total and OVA-specific IgE. In addition, GGTA reduced the expression of TGF-β1 and Smad-3 and lowered the deposition of collagen. To our knowledge, this study was the first to provide experimental evidence that GGTA has antiasthma and antifibrotic effects by reducing airway inflammation and pulmonary fibrosis.

The OVA-induced experimental-asthma model is a well-established animal model that was developed to study the mechanism of asthma and the potential therapeutic efficacy of agents. After OVA sensitization and challenge, mice frequently show symptoms and patterns that are similar to those found in patients with chronic asthma: immediate asthma reactions and infiltration of inflammatory cells (such as eosinophils) into the BALF and lung interstitium [16, 17]. Thus, the experimental model of OVA-induced asthma in the mouse is often used to screen agents with potential antiasthmatic effects.

Chronic asthma is characterized by inflammation of the respiratory tract caused by eosinophil infiltration, T-cell recruitment to the airways, alterations in the Th2 response, and allergen-specific IgE production. Moreover, chronic asthma is accompanied by thickening of the bronchial walls, epithelial damage of the bronchial mucosa, subepithelial fibrosis, increased deposition of the extracellular matrix (ECM) protein collagen, and increased biological activity of cytokines [18]. A dominant Th2 response induces several characteristic features of asthma, including eosinophil recruitment, overproduction of Th2-type cytokines (such as IL-4, IL-5, and IL-13), and elevation in the level of IgE [19]. Activated eosinophils are major contributors to the inflammation that is frequently observed in asthma, which is considered a hallmark of the disease and includes airway epithelial cell loss and damage and airway dysfunction [20]. Th2-type cytokines (such as IL-4, IL-5, and IL-13) and chemokine (eotaxin) have been implicated in the promotion of allergic responses in asthma [21]. IL-4 is a key factor for isotype-switching IgE in B lymphocytes, IL-5 plays a major role in the activation and survival of eosinophils in the airway, and IL-13 is a pleiotropic cytokine that is produced by Th2 cells and other cell types and is involved in eosinophil infiltration into lung tissues [22]. Eotaxin is a potent eosinophil chemoattractant [23], whereas IgE is one of the most important factors in the progression of allergic reactions [24] and has been implicated in the allergic inflammation of asthma, via the promotion of the migration and activation of inflammatory cells. Many studies have reported that the antiasthma effects observed in a mouse model of allergic asthma are exerted via the modulation of its production [22, 25]. Consistent with a previous study, our findings indicate that treatment with GGTA attenuates airway inflammation by reducing the accumulation of inflammatory cells in the airway and lung tissue, the production of Th2 cytokines and eotaxin in BALF, and altering the levels of total and OVA-specific IgE in plasma. These findings were paralleled in a histological examination. Thus, these results demonstrate that GGTA may have potential as an effective anti-inflammatory agent to treat airway inflammation.

Fibrosis is an irreversible change that is present typically in various chronic diseases. In particular, pulmonary fibrosis is characterized by the overproduction of collagen and other ECM components and their accumulation in the lung and other tissues [26]. The pathogenesis of pulmonary fibrosis is very complex and is associated with various types of mediators. Among them, TGF-β1, which is a profibrotic cytokine, is considered to be a key mediator of pulmonary fibrosis and is the most potent inducer of fibroblast activation and ECM synthesis. It also serves as a chemoattractant of monocytes, fibroblasts and mast cells and is correlated with physiological tissue repair and immune regulation [17]. The level of TGF-β1 is elevated in asthma, and its increase is involved in basement-membrane thickness [27]. TGF-β1 regulates collagen via the canonical SMAD pathway by binding to, and activating, specific type I and type II serine/threonine kinase receptors. Smads are a group of intracellular proteins that are critical for transmitting the TGF-β1 signals from the cell surface to the nucleus, to promote the transcription of target genes, such as the genes that encodes collagen [28, 29]. Recent studies revealed that Smad-3 signaling is a key signaling pathway of fibrogenesis in response to many fibrogenic mediators, such as TGF-β1, and is essential for the synthesis of many ECM components, including collagen [30, 31]. Therefore, the TGF-β/Smad signaling pathway plays a key role in the pathogenesis of fibrotic disease. Previous studies demonstrated that the inhibition of pulmonary fibrosis exerts antifibrotic effects via the modulation of the TGF-β1/Smad-3 signaling pathway in repetitive OVA-challenged mice [2, 32]. In our study, OVA-induced mice exhibited pulmonary fibrosis, resulting in the overexpression of TGF-β1/Smad-3 and deposition of collagen. In contrast, treatment with GGTA significantly blocked the pulmonary fibrosis via the inhibition of the release of fibrotic mediators. These findings showed that GGTA modulated the TGF-β1/Smad-3 signaling pathway, which led to collagen deposition and extracellular matrix synthesis. Therefore, GGTA administration may be used as an effective therapeutic agent for a wide range of diseases associated with fibrosis.

Traditional herbal medicines are becoming increasingly popular worldwide for primary health care. Herbal medicines are a mixture of several herbs and are believed to produce synergistic effects, and a reduction of adverse effects and toxicity via herb–herb interactions [33]. Gumiganghwal-tang is a traditional herbal medicine that is composed of a mixture of crude extracts from nine medicinal herbs (Osterici Radix, Saposhnikoviae Radix, Cnidii Rhizoma, Angelicae Dahuricae Radix, Atractylodis Rhizoma, Scutellariae Radix, Rehmanniae Radix Crudus, Asiasari Radix, and Glycyrrhizae Radix et Rhizoma). Previous studies have demonstrated that Osterici Radix [34], Saposhnikoviae Radix [35], and Angelicae Dahuricae Radix [36] possess antiallergic properties; Scutellariae Radix [37] and Rehmanniae Radix Crudus [38] possess anti-inflammatory properties; Cnidii Rhizoma [39] and Asiasari Radix [40] possess antitumor properties; and Atractylodis Rhizoma [41] and Glycyrrhizae Radix et Rhizoma [42] possess antioxidant properties. Hence, we expected that GGTA would exert antiasthmatic and antifibrotic effects based on the pharmacological action of the herb–herb interactions on the OVA-induced chronic asthma model. However, it is difficult to verify the main biological active compound that is responsible for the efficacy of herbal medicine extracts. Although it was demonstrated that GGTA had antiasthmatic and antifibrotic properties, further studies are needed to identify its main active compound.

## Conclusions

Taken together, the findings of our study demonstrated that GGTA significantly attenuated airway inflammation and pulmonary fibrosis by reducing the Th2 cytokines production, accumulation of inflammatory cells and modulating the TGF-β1/Smad-3 signaling pathway. These results suggest that GGTA is effectively protective against repetitive OVA-induced chronic asthma and may be a potential therapeutic agent for allergic asthma involving fibrosis.
